# Liquidity and volatility commonality in the Canadian stock market

**DOI:** 10.1186/s40929-017-0016-9

**Published:** 2017-10-11

**Authors:** Nathan Gold, Qiming Wang, Melanie Cao, Huaxiong Huang

**Affiliations:** 1 Department of Mathematics and Statistics, York University, 4700 Keele St., Toronto, M3J 1P3 Canada; 2 Fields Institute for Research in Mathematical Sciences, 222 College St., Toronto, M5T 3J1 Canada; 3 Present address: Global Risk Management, Scotiabank, 4 King St. W., 14th Floor, Toronto, M5H 1B6 Canada; 4 Finance Area, Schulich School of Business, York University, 4700 Keele St., Toronto, M3J 1P3 Canada

**Keywords:** Canadian stock market, Liquidity, Volatility, Commonality

## Abstract

This paper studies liquidity and volatility commonality in the Canadian stock market. We show that five various liquidity measures display strong evidence of commonality at both market-wide and industry specific levels. Our findings extend the results of previous studies in liquidity commonality, and show that even after controlling for individual determinants of liquidity such as price, volume, and volatility, liquidity commonality remains. In addition to demonstrating liquidity commonality, we also investigated the causal relationship between liquidity and volatility. Our evidence indicates that depth, proportional effective spread, and liquidity changes predict volatility changes for bid-ask spread, depth, and proportional effective spread.

## Introduction

In May 2015, a group at TMX proposed to the participants of the Big Data Industrial Problem Solving Workshop to investigate any commonality and causal relationships between liquidity and volatility of the assets that are traded in the Canadian markets. This workshop was followed by a six-month NSERC Engage project, with a description of the results of project described herein.

It has long been known that liquidity and volatility are correlated components of the market, affecting each other in a multitude of different ways. Liquidity may be defined as the ability of a market participant to quickly buy or sell a given quantity of an asset at any time. Volatility of an asset or market index is measured as the standard deviation of returns for that asset or market index. Beyond traditional correlation analysis between liquidity and volatility, the question of the causal relationship between liquidity changes and volatility changes remains open to debate.

Liquidity commonality has a major impact on market dynamics. By liquidity commonality, we refer to the impact of a market-wide liquidity factor on individual firms, with respect to different liquidity measures. Co-movement in liquidity has far reaching implications in terms of inventory risk and porftolio construction. Under severe conditions, the market may become highly illiquid, tremendously increasing inventory risk, and the ability of market participants to change their positions. Hence, an understanding of liquidity commonality market-wide is needed.

The first work to explore market-wide liquidity commonality was that of Chordia, Roll, Subrahmanyam in 2000 [1]. They determined strong evidence of liquidity commonality amongst 1,169 stocks on the NYSE for the year 2000. The existence of a common liquidity factor for the U.S. market was also confirmed in later papers [2, 3]. Partial evidence of commonality for liquidity for non-U.S. exchanges was also demonstrated by Brockman and Chung in 2002 [4], but comprehensively convincing evidence for liquidity commonality as a global phenomenon was not established until Brockman, Chung, and Pérignon in 2009 [5], who investigated liquidity commonaity for 47 stocks exchanges around the world. With respect to liquidity commonality across different markets — a concept particularly relevant to our considerations — Cao and Wei in 2010 [6] explored liquidity commonality in the options market. They presented convincing evidence of liquidity commonality on both the exchange and options market levels.

Different aspects have been considered with respect to establishing evidence of a causal relationship between liquidity and volatility at an exchange level. From an asset pricing point of view, the effect of liquidity was first argued from a theoretical perspective in 1986 in the seminal paper of Amihub and Mendelson [7], and later by Jacoby, Fowler, and Gottesman in 2000 [8]. More directly, liquidity risk and expected asset returns were considered by Acharya and Pedersen in 2005 [9], who used a capital asset pricing model to understand how liquidity may affect asset prices. Chordia, Sarkar, and Subrahmanyam in 2005 [10] explored how liquidity spillovers from market-capitalisation-based portfolios of the NYSE related to volatility. They determined that both liquidity and volatility changes in one sector are informative in predicting liquidity shifts in another.

The implications of these questions from a practioner’s perspective is significant. Liquidity co-variation is intrinsically tied to inventory risk and information asymmetry [1]. Market-wide shocks in liquidity have not only a within market effect, but also an extra-market international effect [5]. An answer to the question of liquidity and volatility causality will allow more accurate forecasts to be made with respect to the causal variable, improving portfolio performance, as well as risk management practices [7–10]. Understanding exchange and OTC market price dynamics will allow market participants to seek different pricing opportunities.

Motivated by the questions of liquidity commonality, the causal relationship between liquidity and volatility at the exchange level, and the dynamics of price differences between exchange markets and OTC markets, we investigated data from the Toronto Stock Exchange (TSX) data for a period of four years. To examine liquidity commonality, we reproduce the analysis of Chordia, Roll, and Subrahmanyam (2000) [1] focusing for the first time on the Canadian market. We show unambiguous evidence of liquidity commonality in the TSX, even when accounting for time effects, and different market weighting procedures. We consider the effect of industry specific commonality, and show, in agreement with [1] and [5], that industry specific liquidity is a major component of liquidity for a specific firm. We also examine individual components of liquidity, and demonstrate that the Canadian market follows similar patterns to the U.S. market [1] for liquidity determinants.

In order to determine the causal relationship between liquidity and volatility on the TSX, we use an econometric technique known as Granger causality to allow us to determine a directional relationship for the Canadian market [11]. Work of a similar nature using the notion of Granger causality was done by Chordia, Sarkar and Subrahmanyam in 2005 [10], where it was determined the large market capitalisation firms liquidity causes smaller market capitalisation firm volatility. We instead examine the relationship between liquidity and volatility for the same firm, organised by how liquid each firm is. For a large majority of stocks, our findings provide evidence of liquidity changes predicting future changes in volatility.

The rest of our paper is organized into several sections. The Data section describes the data, how it was processed for analysis, and described the liquidity measures we investigate. The Methods section reports our analysis about liquidity commonality, and examines the individual determinants of liquidity and commonality. In the Results and discussion section we report our investigation into the predictive relationship between liquidity and volatility, as well as the OTC and exchange price relationship. In the Conclusion, we summarise our findings and conclude our analysis.

## Data

Exchange transactions and over-the-counter (OTC) data for Toronto Stock Exchange (TSX) stocks were provided by The Canadian Depository for Securities Limited (CDS) from the years 2008 through 2015. The exchange transactions data includes all entries into the TSX, for each trading day, divided into trades and quotes. The trades data subset contains all transaction information such as the name of the security, time-stamp, the transaction price, the shares exchanged, buyer and seller information, and various specialised information such as the trading session, and delivery notes. The quotes data subset contains the name of the security, time-stamp, the bid-price and ask-price, and the bid-size and ask-size.

The OTC or non-exchange trades data set is organised by the day of validation of the transaction, with the security name and International Security Identification Number (ISIN), the transaction currency, the number of shares being exchanged, and the total value of the exchange. The OTC data lacks an individual time of day stamp for when the transaction was validated.

The exchange transaction data was provided in individual zipped.gz files for each trading day. Upon extraction, the data is in the format of a.txt file, with each trade stored as a 60 character long unformatted string, and each quote stored as a 49 character long unformatted string. We developed a Python extraction script to process each data file, and seperate the information strings into comma seperated files to delineate the correct information for each file.

For this report, we decided to focus on the years 2011-2014. These years corresponded to a period of relative stability in the Canadian market, which made it appropriate for investigation and analysis with regard to liquidity, volatility, and price. Due to the nature of the Canadian market, many stocks listed on the TSX do not trade frequently — sometimes only once or twice a month. To avoid data problems such as sparsity and incomplete time series in our analysis, we chose to analyse the firms in the TSX60, an index of 60 large companies traded on the TSX. These stocks are all traded multiple times a day, and represent a significant majority of the largest companies by market capitalisation that are traded on the TSX. This provides us with 996 days of trading observations.

To construct long-term time series of the exchange data, and to smooth out intraday peculiarities in market activity, we used the daily closing activity for each stock. The daily closing data was calculated using an average of the last 30 minutes of market activity for every active trading day. We calculate the following quantities for each transaction [1, 6]; they are used to calculate the five liquidity measures we investigate for each transaction. The quantities are (units in parantheses)

- Trade price *P*_*t*_ ($)
- Ask price *P*_*A*_ ($)
- Bid price *P*_*B*_ ($)
- Bid-Ask midpoint $P_{M} = \frac {1}{2}(P_{A} + P_{B})$ ($)
- Ask size *Q*_*A*_ (Shares)
- Bid size *Q*_*B*_ (Shares)

The same quantities may not be calculated for the OTC data. Since the OTC data contains only the number of shares that are exchanged per transaction, as well as the total value of the transaction, we may only calculate the average trade price per share, and the average volume of shares per day for each firm. As well, the transactions are only listed according to the day, so only a coarse daily time series for each stock may be computed.

### Methods

We consider five liquidity measures, corresponding to every transaction: the bid-ask spread, the proportional bid-ask spread, the quoted depth, the effective bid-ask spread, and the proportional effective bid-ask spread. These are the same liquidity measures considered in past literature [1, 5]. Their acronyms and definitions are given in Panel A of Table 1. These five liquidity metrics provide a comprehensive measurement of liquidity for each firm in the market. The bid-ask spread and proportional bid-ask spread are classical measures of liquidity, with a higher bid-ask spread connotating a greater degree of illiquidity. The quoted depth is a measurement of liquidity in terms of the volume of shares in a trading order; higher depth indicates a more liquid stock. Effective spread and proportional effective spread are devised to measure actual trading costs, as they recognise that many trades occur within the quoted bid-ask spread, and if the proposed transaction volume exceeds the quoted depth, the portion of the order in excess of the quoted depth may be executed at an altered price. As is the case for bid-ask spread, a higher effective spread indicates a more illiquid stock.

**Table 1:** Liquidity variables: definitions and summary statistics

Panel A: Definitions			
Liquidity measure	Acronym	Definition	Units
Bid-Ask Spread	SPR	*P*_*A*_−*P*_*B*_	$
Proportional Bid-Ask Spread	PSRP	(*P*_*A*_−*P*_*B*_)/*P*_*M*_	None
Depth	DEP	$\frac {1}{2}(Q_{A} + Q_{B})$	Shares
Effective Spread	ESPR	2|*P*_*t*_−*P*_*M*_|	$
Proportional Effective Spread	PESPR	2|*P*_*t*_−*P*_*M*_|/*P*_*t*_	None
Panel B: Cross-sectional summary statistics for time series means			
	Mean	Median	Standard deviation
SPR	0.0314	0.0294	0.00952
PSPR	6.13e-4	5.49e-4	2.68e-4
DEP	1727630	1660309	516074
ESPR	0.0390	0.0262	0.2200
PESPR	8.23e-4	5.38e-4	4.76e-4

*P* denotes price and subscripts indicate: *t* = actual transaction, *A* = ask, *B* = bid, *M* = midpoint. *Q* denotes the quanity of shares available for trade for each quote and subscripts indicate: *A* = ask, *B* = bid. We calculate each measure for the average daily closing transactions (final 30 minutes of trading) during the years 2011–2014 using the 60 stocks in the TSX60 index, corresponding to 996 days of trading observations

In Panel B of Table 1, we present cross-sectional summary statistics of the liquidity measures. As anticipated, there is a degree of positive skewness in the daily average spreads and depth; sample means exceed sample medians. We conjecture that one possible explanation of this effect is due to market makers and trading specialists providing the bulk of trading activity, due to their greater access to trading resources than consumer traders. However, it is likely this positive skewness is suggestive of a stable market, where large deviations of the bid-ask spread are simply less likely to occur.

Next we examine the correlations between each of the five liquidity measures and returns in several different cases. We consider the correlations for the TSX60 with equal weighting amongst all market constituents (Table 2 Panel A), the correlations for the TSX60 using market-capitalisation weighting for each constituent (Table 2 Panel B), TD Bank as a representative highly liquid stock (Table 2 Panel C), Barrick Gold Corp. as a representative stock with moderate liquidity (Table 2 Panel D), and finally Saputo Inc. as a stock with low liquidity (Table 2 Panel E). We chose these stocks for each different liquidity level as they represent very different industries, but are also typical of the TSX60 and the Canadian market. Due to the smaller size of the Canadian market, all of the major financial industries in the TSX60 are highly liquid, and indeed make up a fair portion of the highly liquid stocks in the TSX60; we chose TD Bank as a representative. Barrick Gold Corp. is the largest natural resource/materials mining company in the world, and is headquartered in Canada. For this reason, we chose it as a representative moderate liquidity stock. Finally, Saputo Inc. is a food/consumer discretionary company which is fairly illiquid.

**Table 2:** Liquidity measures and returns correlations

	SPR	PSPR	DEP	ESPR	PESPR	Return
Panel A: Correlation of liquidity measures and returns for the TSX60 equal						
SPR	1					
PSPR	0.589	1				
DEP	-0.062	-0.113	1			
ESPR	0.007	0.019	0.009	1		
PESPR	-0.001	0.020	0.007	0.998	1	
Return	-0.072	-0.079	-0.088	-0.3196	-0.3253	1
Panel B: Correlation of liquidity measures and returns for the TSX60 market-cap						
SPR	1					
PSPR	0.665	1				
DEP	0.069	-0.170	1			
ESPR	0.035	0.035	0.026	1		
PESPR	0.019	0.037	0.019	0.986	1	
Return	-0.072	-0.094	-0.095	-0.174	-0.203	1
Panel C: Correlation of liquidity measures and returns for TD bank						
SPR	1					
PSPR	0.749	1				
DEP	-0.387	0.146	1			
ESPR	0.295	0.134	-0.137	1		
PESPR	0.275	0.195	-0.049	0.987	1	
Return	-0.008	-0.068	-0.004	0.034	0.032	1
Panel D: Correlation of liquidity measures and returns for ABX Barrick Gold Corp.						
SPR	1					
PSPR	-0.148	1				
DEP	-.0351	0.548	1			
ESPR	0.003	-0.042	0.004	1		
PESPR	-0.006	-0.018	0.019	0.999	1	
Return	0.040	-0.044	-0.036	-0.210	-0.212	1
Panel E: Correlation of liquidity measures and returns for SAP Saputo Foods						
SPR	1					
PSPR	0.961	1				
DEP	0.037	0.112	1			
ESPR	0.077	0.079	0.029	1		
PESPR	0.078	0.085	0.036	0.999	1	
Return	-0.019	-0.041	0.027	-0.091	-0.092	1

SPR is the quoted spread, PSPR is the proportional spread, DEP is the depth, ESPR is the effective spread, PESPR is the proportional effective spread, and Return is the daily return for each asset. Panel C, Panel D and Panel E display representative stocks for high, medium, and low liquidity, respectively. 60 stocks, years 2011–2014

To begin, we consider the correlatons for the liquidity measures and returns for the TSX60 in both the equally weighted and market-capitalization weighted cases (Table 2 Panel A and Panel B, respectively). As expected, the spread liquidity measures are positively correlated in both cases, and are also both negatively correlated with depth. This refects the inverse relationship between these two measures. The returns are all negatively correlated with liquidity, with the effective spread and proportional effective spread having a 32% and 33%, respectively, negative correlation with market returns in the equally weighted cases of Panel A. However, this is commensurate with the definition of these liquidity metrics, as a larger effective spread signals a greater price discrepancy between quoted bid and ask prices. This suggests that greater liquidity in the market will have a positive effect on returns. Our findings are in agreement with those of Brockman, Chung, and Pérignon in 2009 [5].

We further investigate the correlation amongst the liquidity metrics and returns for three stocks, each representative of those stocks which have high liquidity, moderate liquidity, and low liquidity. The three stocks are TD Bank (Table 2 Panel C), Barrick Gold Corp. (Table 2 Panel D), and Saputo Inc. (Table 2 Panel E), respectively. The results for each firm are representative of the inter-relationship between liquidity measures on an individual firm basis, and highlight the importance of considering each liquidity measure. Interestingly for Barrick Gold Corp., the spread liquidity measures are negatively correlated (-14.8%).

In Table 3 correlations between the aforementioned individual firm liquidity measures and returns with the TSX60 (equally weighted) market liquidity measures and returns are computed. For TD Bank (Panel A), a highly liquid stock, liquidity measures have typically positive and moderate to significant correlations between the stock and the market. Of note is the negative correlation of both TD returns and market returns with nearly every liquidity measure, however the correlation coefficients are fairly small. This demonstrates that even for a highly liquid stock, returns and illiquidity follow opposite trends. These trends also hold true for both Barrick Gold Corp. (Panel B), and Saputo Inc. (Panel C). Interestingly, for each representative stock liquidity, proportional effective spread and market returns demonstrate rather significant negative correlations. This phenomenon suggests that when stocks have a greater differential between trade price and the bid-ask price midpoint, regardless of the current stock price, returns suffer from this loss of liquidity, accordingly.

**Table 3:** Liquidity measures and correlations between high, medium, and low liquidity stocks and TSX60 equally weighted

Panel A: TD (T) and TSX60 (M) liquidity and return correlations												
	T SPR	M SPR	T PSPR	M PSPR	T DEP	M DEP	T ESPR	M ESPR	T PESPR	M PESPR	T Ret	M Ret
T SPR	1											
M SPR	0.094	1										
T PSPR	0.749	0.375	1									
M PSPR	0.533	0.665	0.522	1								
T DEP	-0.387	0.300	0.146	-0.118	1							
M DEP	-0.241	0.069	-0.052	-0.169	0.539	1						
T ESPR	0.295	-0.008	0.135	0.211	-0.137	-0.001	1					
M ESPR	0	0.035	-0.011	0.035	0.005	0.026	0.119	1				
T PESPR	0.275	0.038	0.195	0.232	-0.049	0.035	0.987	0.113	1			
M PESPR	0.011	0.019	-0.007	0.037	-0.005	0.019	0.129	0.986	0.122	1		
T Ret	-0.008	-0.076	-0.068	-0.040	-0.004	-0.059	0.034	0.006	0.032	0.005	1	
M Ret	-0.060	0.007	-0.084	-0.094	-0.044	-0.095	-0.045	-0.174	-0.046	-0.203	0.377	1
Panel B: Barrick Gold Corp. (A) and TSX60 (M) liquidity and return correlations												
	A SPR	M SPR	A PSPR	M PSPR	A DEP	M DEP	A ESPR	M ESPR	A PESPR	M PESPR	A Ret	M Ret
A SPR	1											
M SPR	0.265	1										
A PSPR	0.148	0.303	1									
M PSPR	0.636	0.665	-0.297	1								
A DEP	-0.351	0.003	0.548	-0.329	1							
M DEP	-0.289	0.069	0.380	-0.169	0.626	1						
A ESPR	0.003	0.006	-0.042	0.019	0.004	0.012	1					
M ESPR	0.007	0.035	-0.021	0.035	0.028	0.026	0.950	1				
A PESPR	-0.006	0.010	-0.018	0.011	0.020	0.023	0.999	0.950	1			
M PESPR	0.014	0.019	-0.037	0.037	0.012	0.019	0.988	0.986	0.987	1		
A Ret	0.040	0.007	-0.044	0.002	-0.036	-0.039	-0.210	-0.204	-0.212	-0.210	1	
M Ret	-0.015	0.007	-0.005	-0.094	-0.031	-0.095	-0.221	-0.174	-0.222	-0.203	0.565	1
	S SPR	M SPR	S PSPR	M PSPR	S DEP	M DEP	S ESPR	M ESPR	S PESPR	M PESPR	S Ret	M Ret
S SPR	1											
M SPR	0.198	1										
S PSPR	0.961	0.177	1									
M PSPR	0.498	0.665	0.588	1								
S DEP	0.037	-0.150	0.112	0.010	1							
M DEP	-0.249	0.069	-0.209	-0.169	-0.009	1						
S ESPR	0.077	0.013	0.079	0.054	0.029	0.006	1					
M ESPR	0.029	0.035	0.027	0.035	-0.015	0.026	0.938	1				
S PESPR	0.079	0.014	0.085	0.063	0.036	0.007	0.999	0.938	1			
M PESPR	0.031	0.019	0.032	0.037	-0.009	0.019	0.976	0.986	0.976	1		
S Ret	-0.020	-0.035	-0.041	-0.042	0.027	-0.083	-0.091	-0.092	-0.092	-0.096	1	
M Ret	-0.046	0.007	-0.051	-0.094	0.005	-0.095	-0.219	-0.174	-0.219	-0.203	0.313	1

T is TD Bank (high liquidity), A is Barrick Gold Corp. (medium liquidity) and S is Saputo Inc. (medium liqudity). M represents the equally weighted market variables for liquidity measures and returns. SPR is the quoted spread, PSPR is the proportional spread, DEP is the depth, ESPR is the effective spread, PESPR is the proportional effective spread, and Ret is the daily return for each asset. 60 stocks, years 2011–2014

The market displays vast variability over time for each of the liquidity measures. Table 4 displays summary statistics of daily percentage changes for each of the liquidity measures. Daily percentage changes for each liquidity measure are also positively skewed, with cross-sectional means greater than cross-sectional medians. Consider daily percentage changes in both effective spread and proportional effective spread, which vary day-to-day by nearly 74%. This volatility in effective spreads may be explained by several sources. First, as we report cross-sectional averages, stocks with large effective spreads relative to other stocks will have a significant effect on the average we report. Secondly, the standard deviation of individual mean daily changes is large, suggesting that the variability from stock to stock for effective spread is drastic.

**Table 4:** Absolute daily proportional changes in liquidity variables

	Mean	Median	Standard deviation
|*SPR*|	0.142	0.098	0.157
|*PSPR*|	0.103	0.065	0.127
|*DEP*|	0.268	0.125	0.862
|*ESPR*|	0.742	0.658	1.682
|*PESPR*|	0.728	0.595	1.714

SPR is the quoted spread, PSPR is the proportional spread, DEP is the depth, ESPR is the effective spread, and PESPR is the proportional effective spread. Absolute daily percentage changes represent the proportional change in each liquidity measure across success days, e.g. for liquidity measure *L*_*t*_. 60 stocks, years 2011–2014

After considering correlations between representative stocks in different liquidity tiers and the market, we then analysed the constituent industries of the TSX60 for industry correlation of liquidity measures and returns with the market, using equal weighted market values. The TSX60 is divided into 10 industries, however as some industries contain few stocks, we group some industries together that operate in similar fields. This provides us with a list of eight constituent industries:

- Materials (10.23% market cap weight)
- Consumer Staples (4.97% market cap weight)
- Consumer Discretionary (6.59% market cap weight)
- Energy (21.30% market cap weight)
- Finance (38.87% market cap weight)
- Telecommunications (9.52% market cap weight)
- Industrials (7.61% market cap weight)
- Healthcare (0.91% market cap weight)

Table 5 displays the correlations amongst industries and the market for returns and the liquidity measures. Generally, liquidity measures and returns for each industry have notable correlation with the market. In the case of the finance, energy, and materials industries, the strong correlation amongst returns and effective spread is indicative of their position as large market constituent industries. Interestingly, the bid-ask spread for these larger industries is not as strongly correlated with the market as a whole, as are other industries. This suggests that trading activity on the rest of the market does not have as broad an impact on larger industries. Due to the size of these industries, however, it can be conjectured that this discrepancy is due to these industries operating with a certain degree of autonomy relative to the rest of the market, due to the equal weighting we use for the market.

**Table 5:** Industries and TSX60 liquidity measure correlations

Materials		Cons. Staples		Cons. Discret.		Energy	
Return	0.7422	Return	0.4610	Return	0.6132	Return	0.8330
SPR	0.2767	SPR	0.2877	SPR	0.4740	SPR	0.3653
PSPR	0.1541	PSPR	0.6841	PSPR	0.6931	PSPR	0.7521
DEP	0.9180	DEP	0.2657	DEP	0.3917	DEP	0.7367
ESPR	0.9517	ESPR	0.6052	ESPR	0.9159	ESPR	0.8210
PESPR	0.9877	PESPR	0.8226	PESPR	0.9797	PESPR	0.9554
Finance		Telecomm.		Industrials		Healthcare	
Return	0.7811	Return	0.5970	Return	0.6141	Return	0.3604
SPR	0.3167	SPR	0.9365	SPR	0.5421	SPR	0.5333
PSPR	0.6807	PSPR	0.8449	PSPR	0.4809	PSPR	0.5020
DEP	0.6324	DEP	0.6636	DEP	0.5021	DEP	0.0802
ESPR	0.9520	ESPR	0.5898	ESPR	0.0125	ESPR	0.0169
PESPR	0.9893	PESPR	0.9695	PESPR	0.0223	PESPR	0.0123

Average correlations between liquidity measures and returns for stocks in each of the 10 constituent industries in the TSX60. SPR is the quoted spread, PSPR is the proportional spread, DEP is the depth, ESPR is the effective spread, PESPR is the proportional effective spread, and Return is the daily return for each asset. Cons. Staples is Consumer Staples, Cons. Discret. is Consumer Discretionary, and Telecomm. is Telecommuncations

For smaller industries, specifically industrials and healthcare, the correlation between industry specific effective spreads and market effective spreads is very low. This is likely due to two factors: *(i)* the number of constituent stocks in the industry is very low relative to the overall size of the market, and *(ii)* trading strategies in these sectors do not generally follow principles that guide larger market strategies. Liquidity and returns for the healthcare industry in particular, which contains only one stock — Valeant Healthcare, VRX — will be affected by external information about the firm itself, rather than macroeconomic trends. Hence, with respect to liquidity, these sectors will move in ways asynchronous to the market, rather than with, or opposite, to the market. Trading strategies or portfolio construction techniques based on sector-market correlational structures to offset liquidity risk would not benefit from a hedging perspective including such firms, due to their non-contemporaneous liquidity movements.

## Results and discussion

### Commonality in liquidity measures

Previous literature from Chordia, Roll and Subrahmanyam (2000) [1] and Brockman, Chung, and Pérignon (2009) [5] demonstrate liquidity commonality in the NYSE and in global markets, respectively. While [5] provide evidence of liquidity commonality for the Canadian market, they do not provide an in depth analysis with respect to Canadian market itself. The first question we seek to answer is that of whether or not variation in individual stock liquidity is related to market trading activity after controlling for trading activity in the individual stock itself for the TSX. Variation in liquidity is known to depend on individual stock attributes such as trading volume and price level, but covariation amongst liquidity measures in the Canadian market is at present, unknown. Co-movement in liquidity amongst the market is an essential piece of information for risk managers, as inventory risks and asymmetric information both affect individual stock liquidity. Hence, one may expose herself to significant inventory risk due to periods of high illiquidity if liquidity experiences covariation.

The first stage of our analysis is to construct a simple ‘market model’ time series regression. We regress daily percentage changes in liquidity variables for an individual stock on market measures of liquidity,

1 $$\begin{array}{*{20}l} DL_{j,t} = \alpha_{j} + \beta_{j} DL_{M,t} + \epsilon_{t} \end{array} $$

where *D* *L*_*j*,*t*_, is, for stock *j*, the percentage change (*D*) from trading day *t*−1 to day *t* in liquidity variable *L* (*L* = SPR, PSPR, etc.), *D* *L*_*j*,*t*_=(*L*_*j*,*t*_−*L*_*j*,*t*−1_)/*L*_*j*,*t*−1_, and *ε*_*t*_∼*N*(0,1) is Gaussian or *white* noise. We use percentage changes rather than daily levels because this provides a better representation of how liquidity co-moves. We consider an equally weighted market model, and a market capitalisation weighted market model. The results from this regression using equal weighted market values are reported in Panel A of Table 6. The regression allows a linear relationship to be determined between the changes in market liquidity and changes in individual firm liquidity. Larger values of the regression coefficient, *β*_*j*_, are suggestive of a greater predictive relationship between market liquidity and individual stock liquidity. Indeed, this may be seen explicitly by noting the definition of the estimate of *β*_*j*_,

$$\begin{array}{*{20}l} \hat{\beta_{j}} = \sqrt{\frac{\sigma^{2}_{DL_{M}}}{\sigma^{2}_{DL_{j}}}}\rho(DL_{j},DL_{M}), \end{array} $$

**Table 6:** Market-wide commonality in liquidity for TSX60 equally weighted

	SPR	PSPR	DEP	ESPR	PESPR
Panel A: Concurrent market term commonality					
Coeff. mean	0.192	0.49	1.214	2.989	3.161
Coeff. st. dev.	0.145	0.374	1.236	8.020	8.597
% positive	98.15	100	100	98.18	98.18
% significant	94.44	96.30	98.08	28.85	30.00
Panel B: Concurrent and lagged market term commonality					
Concurrent					
Coeff. mean	0.215	0.542	1.213	2.989	3.161
Coeff. st. dev.	0.162	0.421	1.232	8.021	8.597
% positive	98.15	100	100	100	98.18
% significant	94.44	96.30	98.08	28.85	30.00
Lag					
Coeff. mean	0.061	0.128	-0.017	0.043	0.021
Coeff. st. dev.	0.079	0.0154	0.054	0.110	0.057
% positive	88.89	90.74	38.89	70.91	50.91
% significant	50.00	74.07	5.55	0	0
Panel C: Concurrent, lagged, and lead market term commonality					
Concurrent					
Coeff. mean	0.226	0.575	1.220	2.989	3.161
Coeff. st. dev.	0.169	0.443	1.246	8.022	8.597
% positive	98.15	100	100	98.18	96.36
% significant	94.44	94.44	98.08	28.85	28.00
Lag					
Coeff. mean	0.067	0.145	-0.014	0.043	0.021
Coeff. st. dev.	0.073	0.016	0.050	0.109	0.057
% positive	88.89	92.59	38.89	70.91	50.91
% significant	51.85	77.78	5.55	0	0
Lead					
Coeff. mean	0.023	0.068	0.072	0.026	0.012
Coeff. st. dev.	0.007	0.089	0.159	0.055	0.028
% positive	72.22	85.18	44.44	70.91	63.64
% significant	3.73	31.48	24.07	1.82	0

Time series regression of daily proportional changes of individual stocks liqudity measures on daily proportional changes in equally-weighted average liquidity for all stocks in the TSX60. SPR is the quoted spread, PSPR is the proportional spread, DEP is the depth, ESPR is the effective spread, and PESPR is the proportional effective spread. Daily percentage changes represent proportional changes in each liquidity metric across trading days, e.g. for liquidity measure *L*, the daily change is *Δ* *L*_*t*_=(*L*_*t*_−*L*_*t*−1_)/*L*_*t*−1_. For each individual regression, the TSX60 average does not include the dependent stock’s liquidity measures. Panel A denotes the cross-sectional averages of the regression only with the concurrent market liquidity measure, Panel B contains the cross-sectional average coefficients of the regression with the concurrent market liquidity variable and a lag term, and Panel C contains the cross-sectional average of the regression with the concurrent market liquidity variable, and lead and lag market term. Concurrent, Lag, and Lead denote, respectively, to the same, previous, and next trading day observations of the market liquidity variables. %-positive denotes the percentage of positive regression coefficients, and %-significant denotes the percentage of *t*-statistics greater than the 5% critical level in a one tailed test (+ 1.645). 60 stocks, years 2011–2014

where $\sigma ^{2}_{DL_{M}}, \sigma ^{2}_{DL_{j}}$ are the variances of the daily liquidity measure of the market and stock *j*, respectively, and *ρ* denotes the correlation between *D* *L*_*M*_ and *D* *L*_*j*_. As the residuals are assumed to be normally distributed, the predictive nature of the slope reflects the correlation (indicative of co-moving) amplified by the ratio of the standard deviations (the root of the ratio of a proxy for the volatilities).

When computing the market liqudity measure, *D* *L*_*M*_, that each stock is regressed on, stock *j* is excluded. This is done to remove the potential of a misleading constraint on the average coefficients reported in Table 6. This is especially important for the TSX60 since the number of stocks considered is relatively low in comparison to larger markets around the globe. By removing the constraint, we also remove the opportunity for spurious correlations to corrupt our analysis, since small contributions from each stock can accumulate to a potentially significant total when averaged across all equations.

The results of this stage of the analysis represent impressive evidence of co-movement in liquidity in the TSX60. In Panel A of Table 6, the mean regression coefficient for each liquidity measure using an equally weighted market is displayed. Spread and proportional spread (SPR and PSPR) demonstrate mild and moderate co-movement with the market, respectively, however the percentage of firms with significant tests — that is, firms for which the liquidity measures can be statistically confirmed to co-move with the market — are remarkably high. Traders and risk managers therefore must be cautious with respect to price spreads. With high statistical precision as well, depth (DEP) highly co-moves with the market, suggesting that the bid and ask size of a quote being given for an individual firm may be well approximated by the bid and ask size of quotes for the rest of the market. Thus, inventory risk may be difficult to hedge in the market, since trading activity co-moves across the market.

Our next step was to perform our market model regression, but with a lagged daily percentage change term, *D* *L*_*M*,*t*−1_. This lagged term is the change in the liquidity metric from the previous day, and is used to account for lagged adjustment in commonality. The results of the market model regression with concurrent and lagged terms for equally weighted market variables are presented in Table 6, Panel B,

2 $$\begin{array}{*{20}l} DL_{j,t} = \alpha_{j} + \beta_{j} DL_{M,t} + \gamma_{j} DL_{M,t-1} + \epsilon_{t}. \end{array} $$

While typically positive, the lagged coefficients are small in magnitude, and only in the case of PSPR is the lag coefficient significant. This provides evidence that in most cases, liquidity commonality for individual firms with the market is restricted to the current trading day, and has little ‘spill over’ effect in terms of corrections the next day. The market percentage spread lagged term may be used as an imprecise predictor of current individual stock percentage spread, however commonality is far greater for the concurrent market.

For the final stage of the market model regression, we include a lead daily percentage change term, *D* *L*_*M*,*t*+1_, or the next day’s change in market liquidity measure. This lead term serves as a proxy for the next day’s market wide liquidity, and may be used as an expected value for liquidity on the following day. This particular term is relevant for portfolios constructed with respect to trading on liquidity, as one may seek to hedge liquidity risk the following day, or change current trading strategies based on the expected market liquidity effect the proceeding day. The calculated mean *β*, *γ*, and *ζ* coefficients, for concurrent, lagged, and lead daily market percentage changes for equally weighted market variables are presented in Table 6, Panel C,

3 $$\begin{array}{*{20}l} DL_{j,t} = \alpha_{j} + \beta_{j} DL_{M,t} + \gamma_{j} DL_{M,t-1} + \zeta_{j}DL_{M,t+1} + \epsilon_{t}. \end{array} $$

The concurrent mean regression coefficients remain largely unchanged, upon inclusion of the lagged and lead variables. The spread and proportional spread increase marginally, whereas the other liquidity measures do not vary a great deal. Thus, co-movement amongst liquidity is dominated by concurrent (same day) market liquidity changes, rather than adjustments from the previous day, or projections of future liquidity changes. These results are in good agreement with existing literature [1, 4, 5, 12].

We report the results of our market model regression using market capitalisation weighted variables in Table 7. The average regression coefficients for the concurrent market model — equation (1) — are presented in Panel A. While spread, proportional spread, and depth remain relatively similar to their equally weighted counterparts, this does not hold for the effective spreads. For effective spread (ESPR) and proportional effective spread (PESPR), the regression coefficients are markedly reduced, along with their standard deviations. This pattern is the precise opposite of market model regressions that involves returns; return coefficients are typically smaller for equally weighted markets, as smaller stocks will be more sensitive to the market. Contradicting this trend, the results we find here demonstrate that smaller stocks are less sensitive to market-wide shocks in spread, and their trading prices will be less sensitive to total market movements. Smaller stocks by market capitalisation may then be used in hedging portfolios to protect against market shocks in spread.

**Table 7:** Market-wide commonality in liquidity for TSX60 market capitalisation weighted

	SPR	PSPR	DEP	ESPR	PESPR
Panel A: Concurrent market term commonality					
Coeff. mean	0.371	0.685	1.226	1.641	1.523
Coeff. st. dev.	0.289	0.547	1.207	4.440	4.141
% positive	98.15	100	100	100	100
% significant	94.44	94.44	98.04	26.00	28.00
Panel B: Concurrent and lagged market term commonality					
Concurrent					
Coeff. mean	0.406	0.736	1.225	1.641	1.523
Coeff. st. dev.	0.322	0.598	1.203	4.440	4.141
% positive	98.15	100	100	100	100
% significant	94.44	94.44	98.08	26.00	28.00
Lag					
Coeff. mean	0.094	0.141	-0.015	0.009	0.004
Coeff. st. dev.	0.109	0.0165	0.050	0.026	0.013
% positive	87.04	88.89	40.74	60.00	58.18
% significant	66.67	70.37	3.70	0	0
Panel C: Concurent, lagged, and lead market term commonality					
Concurrent					
Coeff. mean	0.424	0.767	1.232	1.641	2.136
Coeff. st. dev.	0.334	0.623	1.216	4.441	5.868
% positive	98.15	100	100	100	100
% significant	94.44	94.44	98.04	26.00	28.00
Lag					
Coeff. mean	0.104	0.159	-0.013	0.009	0.011
Coeff. st. dev.	0.111	0.175	0.047	0.026	0.029
% positive	90.74	88.89	40.74	63.63	65.45
% significant	68.52	72.22	3.70	0	0
Lead					
Coeff. mean	0.041	0.071	0.072	0.009	0.009
Coeff. st. dev.	0.068	0.0100	0.154	0.018	0.179
% positive	88.33	87.04	44.44	69.09	69.09
% significant	18.52	35.19	27.78	0	0

Time series regression of daily proportional changes of individual stocks liqudity measures on daily proportional changes in market capitalisation weighted average liquidity for all stocks in the TSX60. SPR is the quoted spread, PSPR is the proportional spread, DEP is the depth, ESPR is the effective spread, and PESPR is the proportional effective spread. Daily percentage changes represent proportional changes in each liquidity metric across trading days, e.g. for liquidity measure *L*, the daily change is *Δ* *L*_*t*_=(*L*_*t*_−*L*_*t*−1_)/*L*_*t*−1_. For each individual regression, the TSX60 average does not include the dependent stock’s liquidity measures. Panel A denotes the cross-sectional averages of the regression only with the concurrent market liquidity measure, Panel B contains the cross-sectional average coefficients of the regression with the concurrent market liquidity variable and a lag term, and Panel C contains the cross-sectional average of the regression with the concurrent market liquidity variable, and lead and lag market term. Concurrent, Lag, and Lead denote, respectively, to the same, previous, and next trading day observations of the market liquidity variables. %-positive denotes the percentage of positive regression coefficients, and %-significant denotes the percentage of *t*-statistics greater than the 5% critical level in a one tailed test (+ 1.645). 60 stocks, years 2011–2014

Panel B of Table 7 contains the results of the concurrent and lagged term regression for market capitalisation weighted market variables. As in the case for equally weighted variables, the lagged terms are positive and statistically significant, yet small in magnitude. The results of the market regression model with concurrent, lagged, and lead terms are reported in Panel C of Table 7. Interestingly, the concurrent regression coefficient for the percentage effective spread increases quite notably when considering market weight, although the lag and lead terms do not contribute even mild predictive power.

For a deeper analysis of sources of liquidity commonality, we constructed a ‘market and industry model’ regression. Our model used both market *D* *L*_*M*,*t*_ and industry liquidity measures, *D* *L*_*I*,*t*_, equally weighted,

4 $$\begin{array}{*{20}l} DL_{j,t} = \alpha_{j} + \beta^{M}_{j} DL_{M,t} + \gamma^{M}_{j} DL_{M,t-1} + \zeta^{M}_{j}DL_{M,t+1} + \beta^{I}_{j} DL_{I,t}& \\ + \gamma^{I}_{j} DL_{I,t-1} + \zeta^{I}_{j}DL_{I,t+1}& \end{array} $$

where we have included lag and lead daily percentage change, *D* *L*_*I*,*t*−1_,*D* *L*_*I*,*t*+1_, terms as well. Regression coefficients are delineated by $\beta _{j}^{M}$ for market, and $\beta _{j}^{I}$ for industry (the lag and lead variable coefficients follow the same convention). We follow the same practice of removing firm *j* when computing the industry average.

We report the mean regression coefficients and standard deviations in Table 8. With the exception of effective spread, liquidity is influenced by both the market, and the industry specific to the firm. For the spread, proportional spread, depth, and proportional effective spread, we see that the industry component is larger than the market component. Thus, covariation in liquidity can be determined by market variation, and to a much larger degree, industry specific movement. According to our findings, trading activity and volatility exhibit greater intra-industry commonality than market commonality. Inventory risk, therefore, is more likely an industry specific phenomenon, than a market wide phenomenon, although both components must be considered.

**Table 8:** Market wide and industry specific commonality in liquidity

	SPR		PSPR		DEP		ESPR		PESPR	
	Market	Industry	Market	Industry	Market	Industry	Market	Industry	Market	Industry
Concurrent										
Coeff. mean	0.119	0.517	0.321	0.628	0.144	0.761	1.445	0.272	1.051	2.196
Coeff. std. dev.	0.116	0.675	0.327	0.662	0.848	0.767	7.190	0.845	7.577	7.045
Lag										
Coeff. mean	0.019	0.093	0.042	0.119	-0.038	0.037	0.205	-0.042	-0.012	0.277
Coeff. std. dev.	0.105	0.163	0.206	0.194	0.096	0.056	1.252	1.015	1.264	1.647
Lead										
Coeff. mean	0.002	0.056	0.009	0.061	0.022	0.021	0.102	-0.0132	-0.002	0.116
Coeff std. dev.	0.034	0.100	0.082	0.141	0.173	0.095	1.020	0.845	1.217	1.376

Time series regression of daily proportional changes of individual stocks liqudity measures on daily proportional changes in equally-weighted average liquidity for all stocks in the TSX60 (Market) and sample stock in the same industry (Industry). SPR is the quoted spread, PSPR is the proportional spread, DEP is the depth, ESPR is the effective spread, and PESPR is the proportional effective spread. Daily percentage changes represent proportional changes in each liquidity metric across trading days, e.g. for liquidity measure *L*, the daily change is *Δ* *L*_*t*_=(*L*_*t*_−*L*_*t*−1_)/*L*_*t*−1_. For each individual regression, the TSX60 average and Industry average do not include the dependent stock’s liquidity measures. Concurrent, Lag, and Lead denote, respectively, to the same, previous, and next trading day observations of the market liquidity variables

The negligible effective spread regression coeffient may be explained by the definition of industries in the Canadian market. As some of the industries we consider may contain only one stock, and occupy a small percentage of overall market capitalisation relative to the market, their industry specific movement likely has less impact than overall market movement, when considered in our analysis.

### Commonality and individual determinants of liquidity

To complete our analysis of commonality of liquidity measures, we examine how individual trading volume, volatility, and price are influential in determining volatility. Economically, trading volume should reduce spreads, increasing liquidity and depth, while volatility should increase spreads, reducing liquidity. If informed traders earn greater profit when volatility is higher, as they may use privileged information and greater access to resources to capitalise on market volatility, then spreads should increase in response.

Market price should have a broad influence on spreads; lower priced stocks will not have the same bid-ask spread as higher priced stocks. Depth should decrease with price, while the proportional spreads should not be influenced by price, as they are constructed to alleviate this constraint. To support this, we performed the following multiple regression,

5 $$\begin{array}{*{20}l} L_{j,t} = \alpha_{j} + \beta_{j}{S}_{j,t} + \gamma_{j}{P}_{j,t} + \zeta_{j}{V}_{j,t} + IL_{j,t} + \epsilon_{t} \end{array} $$

where *L*_*j*,*t*_ is the individual stock liquidity measure, *S*_*j*,*t*_ is the volatility for the individual stock, *P*_*j*,*t*_ is the stock price, *V*_*j*,*t*_ is the trade volume for the individual stock, and *I* *L*_*j*,*t*_ is the industry specific liquidity measure.

Table 9 displays the separate marginal influences on liquidity of individual attributes volatility, price, and trading volume, as well as commonality measured by industry liquidity. As expected, individual volatility (STD in Table 9) has a positive effect on spread liquidity measures, and negative effect on depth liquidity measure. As well, individual trading volume has a negative effect on spreads, and a positive effect on depth. The impacts are fairly large for each measure, suggesting that individual determinants of liquidity are important to consider when investigating the effect of liquidity risk.

**Table 9:** Individual liquidity determinants and industry commonality

	SPR	PSPR	DEP	ESPR	PESPR
STD	0.151	0.122	-0.056	0.362	0.306
Price	0.094	-0.583	-0.204	0.109	-0.510
Volume	-0.168	-0.119	0.483	-0.165	-0.110
Industry	0.470	0.508	0.510	0.485	0.546

Cross-sectional regression of daily individual stock liquidity measures on individual daily returns from the preceeding month (STD), the concurrent day’s mean prices level (Price), the day’s dollar trading volume (Volume), and equally-weighted liquidity measure of all the stocks in the same industry (Industry). SPR is the quoted spread, PSPR is the proportional spread, DEP is the depth, ESPR is the effective spread, and PESPR is the proportional effective spread. The cross-sectional regression coefficients are averaged across the 996 trading days

Industry liquidity remains to be a strong influence on individual stock liquidity, even when considering other determinants such as volatility, trading volume, and stock price. This provides further evidence of commonality as an essential characteristic of liquidity in the market, as noted in [1, 6].

Interestingly, price has a pronounced negative effect on proportional effective spread. At present, there exists no hypotheses to explain this finding, other than discreteness. As a minimum quote spread must be satisfied in order for a trade to be eligible to occur, stocks liquid enough to trade at the minimum spread would exhibit negative correlation between price and the percentage spread. Only for stocks which have a high enough price, or are illiquid enough to have quotes greater than the minimum spread would this not be a problem.

### Volatility, liquidity and pricing causality

Beyond the question of determining commonality amongst liquidity measures in the Canadian market, we are also interested in establishing a causal directional relationship between measures of liquidity and volatility, both on the exchange, and between the OTC market and the exchange. Directional relationships between liquidity and volatility reveal a way to predict how volatility or liquidity shocks will effect one another, as well as predicting upcoming trends in the dependent variable by using current trends in the causal variable. This would help to extend the findings of [10], who determined that liquidity changes in large market capitalisation stocks causes changes in volatility for small market capitalisation stocks, by providing the relationship for individual stocks.

To establish causal relationships between liquidity and volatility, we use the econometric notion of Granger causality [11]. Variable *X* is said to *Granger cause* variable *Y* if predictions of variable *Y* are more accurate if lagged information of variable *X* is included along with lagged information of variable *Y*. We compute vector autoregressive (VAR) models of each liquidity measure and volatility for each stock to predict future values of each variable. The VAR model for liquidity measures, *L*, with volatility included, *VOL*, is given by,

6 $$\begin{array}{*{20}l} L_{j,t} = \sum_{k=1}^{p}A_{LL,k}L_{t-k} + \sum_{k=1}^{p}A_{LVOL,k}VOL_{t-k} + \epsilon_{L,t} \end{array} $$

where *A*_*LL*,*k*_ and *A*_*LVOL*,*k*_ are the coefficient matrices of the autoregressive model used for forecasting, *k* is the number of lagged terms included in the prediction of future values of the variable, and *ε*_*L*,*t*_ is random noise from external sources. The VAR model for volatility, *VOL*, with liquidity measures, *L*, included is,

7 $$\begin{array}{*{20}l} VOL_{j,t} = \sum_{k=1}^{p}A_{VOLVOL,k}VOL_{t-k} + \sum_{k=1}^{p}A_{VOLL,k}L_{t-k} + \epsilon_{VOL,t}. \end{array} $$

To test whether or not one variable Granger causes the other, we perform a statistical hypothesis test known as an *F*-test to see if there is a significant difference in the accuracy of predictions for the variable with the potential causal factor, and without. That is, we check to see if the VAR models given by equations (4) and (5) are more accurate than VAR models without the other variable,

8 $$\begin{array}{*{20}l} L_{j,t} &= \sum_{k=1}^{p}A_{LL,k}L_{t-k} + \epsilon_{L,t} \end{array} $$

9 $$\begin{array}{*{20}l} VOL_{j,t} &= \sum_{k=1}^{p}A_{VOLVOL,k}VOL_{t-k} + \epsilon_{VOL,t}. \end{array} $$

The results of the Granger causality tests between liquidity measures and volatility for the TSX60 on the exchange are presented in Table 10 with a one day lagged term. Since there are several factors which may influence liquidity and volatility separately, not every stock will display a significant causal relationship between the two; for example, bid-ask spread (SPR) has 80% of stocks which have a significant predictive causal relationship between bid-ask spread, where as only 55% of stocks have such a predictive causal relationship for depth (DEP). We attempted longer lag terms, however the results do not vary, so we omit their presentation.

**Table 10:** Granger Causality between liquidity measures (Liq.) and volatility (Vol.) Granger causality regression between liquidity measures for individual stocks and the volatility of their asset prices with a one day lagged term for the constituent stocks of the TSX60

	Vol. −> Liq.	Liq. −> Vol.	% Significant results
SPR	11	37	80.00
% of significant	22.92	77.1	
PSPR	7	28	58.33
% of significant	20	80	
DEP	11	22	55.00
% of significant	33.33	66.67	
ESPR	26	24	83.33
% of significant	52	48	
PESPR	15	24	65.00
% of significant	38.46	61.54	

We test if liquidity Granger causes volatility (Liq. → Vol.) or if volatility Granger causes liquidity (Vol. → Liq.). We report the total number of significant extreme F-test statistics for each test direction. %-Significant results denotes the percentage of *t*-statistics greater than the 5% critical level in a one tailed test (+ 1.645). SPR is the quoted spread, PSPR is the proportional spread, DEP is the depth, ESPR is the effective spread, and PESPR is the proportional effective spread

Our calculations display a clear trend of liquidity predicting causing volatility for exchange traded stocks in the TSX60. In particular, for all liquidity measures except effective spread, including liquidity in future predictions of volatility improves prediction accuracy to a significant degree. Even for effective spread, the number of firms for which changes in volatility can be said to predict changes in liquidity only exceeds the opposite relationship by 4%. Hence, we can deduce that liquidity is a determining factor of price volatility, and should be included when modelling and forecasting volatility.

Likely of most interest to market makers and traders is the significant predictive relationship between spreads and price volatility. A trader may use her current knowledge of the spread for a particular stock, portfolio, or even industry to make better predictions of future shifts in volatility. This in turn may be used to re-weight portfolios to either protect against, or capitalise on, future shifts in volatility, and also improve risk management practises with respect to these predictions.

Trading activity provides one possible explanation for this relationship of liquidity for volatility. Traders observing narrower bid-ask spreads (greater liquidity) for specific stocks, and more generally for the market overall, will react by actively trading the stock, increasing the volume of quotes, and therefore the volume of trading. This will in turn cause the price to move according to the increased trade demand, creating increased volatility in the share price. Conversely, when the bid-ask spread widens (low liquidity), trading activity will slow down, causing share prices to become more stable. There is also evidence of bidirectionality with this predictive relationship, as we note in Table 10. For the effective spread liquidity measure a narrow majority of stocks exhibit a reverse predictive relationship with volatility; that is, volatility changes being predictive of changes in effective spread. The nature of this relationship remains a focus of future work.

## Conclusion

The dynamics of the Canadian market with respect to liquidity and volatility are multifaceted. We have extended the work of [1] and [5] to focus on commonality in liquidity for the Canadian market. Liquidity changes are common across the market, and even more so within specific industries. We have demonstrated that market and industry specifc liquidity factors have a predominant effect on the liquidity of individual assets. Hence, market- and industry-wide shocks in liquidity will have pronounced effects of the liquidity of individual assets.

This provides an interesting piece of information for practitioners concerned about inventory risk in the Canadian market. To enter (exit) a position, one must ensure that there is sufficient liquidity to buy (sell) the stock on the market. As stocks share both a common market and industry liquidity factor, a market participant potentially exposes themselves to a large downside, since the required inventory may not be present in the market to capitalise on a certain position. Consider also our findings with respect to individual determinants of liquidity and industry commonality. We have shown that volatility has a positive influence on spreads, and a negligible negative influence on quote depth. Most interestingly, the price of the stock has a negative influence on proportional spreads. This question remains to be resolved. Despite the other influences of individual liquidity determinants, the industry factor representing commonality remains to be a significant and positive influence on liquidity for individual firms; commonality is a prototypical characteristic of liquidity.

For the majority of constituent firms of the TSX60 index, liquidity is a driving predictive factor of volatility in price and returns. Under typical trading activity, liquidity will first change, resulting in a change in volatilty. As we discussed, for the price to be able to move, there must be a sufficient volume of stock to trade, as well as a sufficiently low bid-ask spread.

A thought-provoking avenue for future work would be to research the effect of market volatility and liquidity on OTC prices. As we have shown that liquidity drives volatility on the exchange, it would be illuminating to examine whether or not the same relationships exist between markets. Due to the different nature of the OTC market, it would also be interesting to investigate if commonality exists in liquidity for the OTC market. Since the OTC market is mostly made up of bi-lateral trading activity, liquidity commonality in this market would provide a unique view toward information asymmetry, and its effect on pricing.
